# RASSF1A Regulates Spindle Organization by Modulating Tubulin Acetylation via SIRT2 and HDAC6 in Mouse Oocytes

**DOI:** 10.3389/fcell.2020.601972

**Published:** 2020-10-26

**Authors:** Hyuk-Joon Jeon, Jeong Su Oh

**Affiliations:** Department of Integrative Biotechnology, College of Biotechnology and Bioengineering, Sungkyunkwan University, Suwon, South Korea

**Keywords:** oocyte, spindle organization, tubulin acetylation, SIRT2, HDAC6, RASSF1A

## Abstract

Dynamic changes in microtubules during cell cycle progression are essential for spindle organization to ensure proper segregation of chromosomes. There is growing evidence that post translational modifications of tubulins are the key factors that contribute to microtubule dynamics. However, how dynamic properties of microtubules are regulated in mouse oocytes is unclear. Here, we show that tumor suppressor RASSF1A is required for tubulin acetylation by regulating SIRT2 and HDAC6 during meiotic maturation in mouse oocytes. We found that RASSF1A was localized at the spindle microtubules in mouse oocytes. Knockdown of RASSF1A perturbed meiotic progression by impairing spindle organization and chromosome alignment. Moreover, RASSF1A knockdown disrupted kinetochore-microtubule (kMT) attachment, which activated spindle assembly checkpoint and increased the incidence of aneuploidy. In addition, RASSF1A knockdown decreased tubulin acetylation by increasing SIRT2 and HDAC6 levels. Notably, defects in spindle organization and chromosome alignment after RASSF1A knockdown were rescued not only by inhibiting SIRT2 or HDAC6 activity, but also by overexpressing acetylation mimicking K40Q tubulin. Therefore, our results demonstrated that RASSF1A regulates SIRT2- and HDAC6-mediated tubulin acetylation for proper spindle organization during oocyte meiotic maturation.

## Introduction

Microtubules are cylindrical cytoskeletal polymers composed of α/β-tubulin dimers and are implicated in a variety of cellular processes, including cellular transport, maintenance of cell structure, and spindle formation and chromosome segregation during cell division (Desai and Mitchison, 1997). Microtubules are highly dynamic by making a rapid change between growing and shrinking states. Although these dynamic properties are essential for various cellular functions, it remains unclear how microtubule dynamics are precisely controlled within cells. However, a growing body of evidence indicates that post-translational modifications of tubulins are key factors that contribute to microtubule dynamics (Magiera and Janke, 2014; Strzyz, 2016). Acetylation of lysine 40 (K40) of α-tubulin is one of the most-studied post-translational modifications that alters the microtubule structure and affects the interactions between microtubules and microtubule-associated proteins (Sadoul and Khochbin, 2016). K40 acetylation in α-tubulin is catalyzed by the acetyltransferase αTAT, and deacetylation is performed by the deacetylases HDAC6 and SIRT2 (Hubbert et al., 2002; North et al., 2003; Zhang et al., 2014; Coombes et al., 2016). Altered level of K40 acetylation has been shown to correlate with various disease, such as cancer, neurologic pathologies, heart diseases, inflammation, and viral infections (Li and Yang, 2015). However, precise knowledge of the physiological function of K40 acetylation in α-tubulin remains elusive.

In mammalian oocytes, dynamic reorganization of microtubules is essential for establishing a bipolar spindle, which orchestrates chromosome alignment and segregation during oocyte meiosis (Bennabi et al., 2016). Defects in this process cause erroneous attachment of kinetochores to microtubules, which increases the likelihood of chromosome missegregation and aneuploidy (Thompson and Compton, 2011). However, a surveillance mechanism, spindle assembly checkpoint (SAC), monitors erroneous kinetochore-microtubule (kMT) attachments, and prevents anaphase onset until all chromosomes are stably attached to spindle microtubules (Shah and Cleveland, 2000; Taylor et al., 2004).

Ras-association domain family 1A (RASSF1A) is a recently discovered tumor suppressor whose inactivation is implicated in development of many human cancers (Dammann et al., 2000; Burbee et al., 2001; Chan et al., 2003; Pan et al., 2005; Pfeifer and Dammann, 2005). Numerous studies have shown that RASSF1A appears to regulate multiple biological processes. For instance, overexpression of RASSF1A promotes apoptosis, cell cycle arrest, and decrease of tumorigenicity of cancer cell lines. In contrast, RASSF1A downregulation causes loss of cell cycle control, enhanced genetic instability, enhanced cell motility, and resistance to apoptosis (Donninger et al., 2007). Although the mechanisms behind these activities remain under investigation, emerging evidence suggests that the function of RASSF1A is associated with microtubules (Liu et al., 2003; Dallol et al., 2004; Vos et al., 2004). Indeed, RASSF1A has been shown to colocalize with microtubules and promote their stabilization (Liu et al., 2003; Dallol et al., 2004; Song et al., 2004; Vos et al., 2004). Moreover, overexpression of RASSF1A protein protects microtubules against depolymerizing agents like nocodazole, whereas RASSF1A mutants have impaired ability to bind and acetylate microtubules (Dallol et al., 2004; Vos et al., 2004; Jung et al., 2013). The underlying mechanisms of RASSF1A associated with microtubules remain elusive, but it is obvious that RASSF1A has the capacity to profoundly influence microtubule dynamics both positively and negatively.

Although several studies have demonstrated prevalent roles of RASSF1A in regulation of microtubule dynamics, its role in oocyte maturation has not been explored. In this study, we investigated the functions of RASSF1A during meiotic maturation in mouse oocytes. We found that RASSF1A is localized at spindle microtubules and regulates spindle organization and chromosome segregation by modulating tubulin acetylation via SIRT2 and HDAC6 in mouse oocytes.

## Materials and Methods

### Oocyte Collection and Culture

Female 3-week-old ICR mice (Koatech, Korea) were used in all experiments. Experiments were approved by the Institutional Animal Care and Use Committees of Sungkyunkwan University (approval ID: SKKUIACUC2019-04-28-4). Ovaries were isolated from mice 46–48 h after injection of 5 IU pregnant mare serum gonadotropin (PMSG). Fully grown cumulus-enclosed oocytes were collected from follicles and cultured in M2 medium supplemented with 200 μM 3-isobutyl-1-methylxanthine (IBMX) to prevent meiotic resumption. For *in vitro* maturation, oocytes were cultured in IMBX-free M2 medium under mineral oil at 37°C in a 5% CO_2_ incubator. For analysis of kMT attachment, oocytes at MI stage were cultured in 4°C M2 medium for 10 min. For chemical treatment, oocytes were treated with 20 μg/ml nocodazole, 10 μM taxol, 2 μM AZ3146 (Selleck Chemicals), 5 μM AGK2, or 2 μM tubacin. All chemicals and culture media were purchased from Sigma-Aldrich unless stated otherwise.

### Plasmid Construction and mRNA Synthesis

The RASSF1A and tubulin K40Q clones were obtained from Addgene (RASSF1A, #37016; tubulin K40Q, #32912). The full-length cDNA sequence encoding RASSF1A was subcloned into pRN3-mCherry vector and *in vitro* transcribed using a mMessage mMachine kit (Ambion). Tubulin K40Q clone was directly *in vitro* transcribed and polyadenylated using mMessage mMachine kit and poly(A) tailing kit (Ambion), respectively.

### Microinjection

Two different siRNAs targeting RASSF1A were designed and purchased from local company (Bioneer, Korea) and diluted in RNase-free water with a final 50 μM concentration. The sequences of RASSF1A siRNAs were CUGAACGGCAUGGCCAAGU (#56289-1) and CCUCCUCU AAGGGAAAGGU (#56289-2). Approximately 5–10 pl of siRNA or cRNA was microinjected into the cytoplasm of oocytes using a FemtoJet microinjector (Eppendorf, Germany) with a Leica inverted microscope (DMIRB) equipped with a micromanipulator (Narishige, Japan). Control oocytes were microinjected with AccuTarget Control siRNA (SN-1003; Bioneer, Korea). After injection, oocytes were cultured for 24 h in medium containing IMBX. The oocytes were then transferred to fresh medium and cultured under mineral oil at 37°C in an atmosphere of 5% CO_2_ in air.

### Immunostaining

Oocytes were fixed in 4% paraformaldehyde for 20 min and permeabilized in phosphate buffered saline (PBS) with 0.25% Triton X-100 for 30 min. After permeabilization, oocytes were blocked in 3% BSA in PBS for 1 h at room temperature. Oocytes were incubated overnight at 4°C with primary antibodies and then at room temperature for 2 h with secondary antibodies. Chromosomes were counterstained with DAPI. Oocytes were examined under a confocal laser-scanning microscope (LSM 700; Zeiss, Germany) equipped with a C-Apochromat 40×/1.2 water immersion objective. ZEN LSM software (Zeiss, Germany) was used to measure and analyze the intensity of fluorescence. Primary antibodies for immunostaining were anti-RASSF1A antibody (Abcam, ab23950, 1:100), anti-acetylated-α-tubulin (acetyl-K40) antibody (Sigma, T7451, 1:500; Abcam, ab179484, 1:500), anti-BubR1 antibody (Abcam, ab28193, 1:100), and anti-centromere antibody (Antibodies Incorporated, 15-234, 1:100). Secondary antibodies were Alexa Fluor 488-conjugated anti-sheep antibody (Abcam, ab150177, 1:500), Alexa Fluor 594-conjugated anti-rabbit antibody (Jackson ImmunoResearch, 111-585-144, 1:500), Alexa Fluor 488-conjugated anti-mouse antibody (Jackson ImmunoResearch, 115-545-144 1:500), Alexa Fluor 594-conjugated anti-mouse antibody (Jackson ImmunoResearch, 111-585-146, 1:500), and Alexa Fluor 488-conjugated anti-rabbit antibody (Jackson ImmunoResearch, 115-545-144 1:500).

### Chromosome Spreading

Oocytes were exposed to acidic Tyrode’s solution (pH 2.5) for 1 min to remove the zona pellucida. After brief recovery in fresh medium, the oocytes were fixed in 1% paraformaldehyde in distilled water (pH 9.2) containing 0.15% Triton X-100 and 3 mM dithiothreitol. The slides were dried slowly in a humid chamber for several hours, and then blocked with 1% BSA in PBS for 1 h at room temperature. Oocytes were incubated with a primary antibody overnight at 4°C and then with a secondary antibody for 2 h at room temperature. DNA was stained with DAPI, and the slides were mounted for observation by confocal microscope.

### Immunoblotting Analysis

Oocytes were lysed in SDS sample buffer at 95°C for 8 min and subjected to SDS-PAGE. Samples were transferred to PVDF membranes, and blocked in TBST (TBS containing 0.5% Tween 20) with 1% BSA for 1 h at room temperature. Membranes were incubated with primary antibodies overnight at 4°C. After three washes in TBST, membranes were incubated with secondary antibodies for 2 h at room temperature. The blots were developed with the ECL Plus Western blotting detection kit (GE Healthcare). Primary antibodies for immunoblotting were anti-RASSF1A antibody (Abcam, ab23950, 1:500), anti-acetylated-α-tubulin (acetyl K40) antibody (Sigma, T7451, 1:500), anti-α-tubulin antibody (Abcam, ab7291, 1:500), anti-HDAC6 antibody (Cell Signaling, #7612, 1:500), anti-SIRT2 antibody (Abcam, ab67299, 1:500) and anti-β-actin antibody (Cell Signaling, #4967, 1:2,500). Secondary antibodies were HRP-conjugated anti-rabbit or anti-mouse antibodies (Jackson ImmunoResearch, 111-035-144, 111-585-144, 1:2,500).

### Statistical Analysis

Statistical analysis was performed with GraphPad Prism 5.0 (GraphPad Software Inc.). The data are presented as the mean ± SEM of at least three independent experiments unless otherwise stated. Differences between two groups were analyzed by Student’s *t*-test, and comparisons between more than two groups were analyzed by one-way ANOVA with Tukey’s *post-hoc* test. *P* < 0.05 was considered statistically significant.

## Results

### Expression and Localization of RASSF1A During Meiotic Maturation in Mouse Oocytes

**FIGURE 1:**
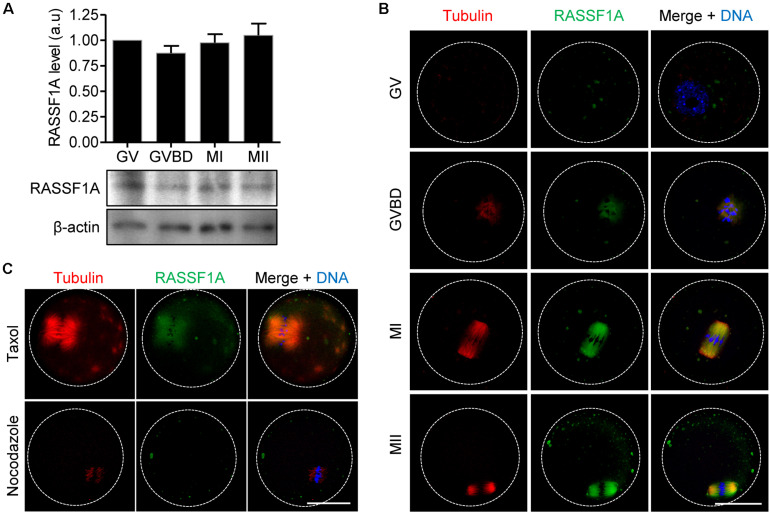
Expression and localization of RASSF1A during meiotic maturation in mouse oocytes. **(A)** Oocytes at GV, GVBD, MI, and MII stages were collected and subjected to immunoblot analysis with anti-RASSF1A antibody. β-actin was used as a loading control. Each lane contains 50 oocytes. Normalized expression of RASSF1A was quantified and expressed as the mean ± SEM from two independent experiments. **(B)** Immunostaining of RASSF1A during oocyte meiotic maturation. Oocytes at different stages were fixed and stained with anti-RASSF1A antibodies. DNA and spindle were stained with DAPI and anti-acetylated-α-tubulin antibody, respectively. Dashed line indicates oocyte cortex. Scale bar, 40 μM. **(C)** Oocytes at the MI stage were cultured in medium containing 10 μM taxol for 45 min or 20 μg/ml nocodazole for 10 min and then stained with anti-RASSF1A antibody. DNA and spindle were stained with DAPI and anti-acetylated-α-tubulin antibody, respectively. Dashed line indicates oocyte cortex. Scale bar, 40 μM.

We first examined the expression of RASSF1A during meiotic maturation in mouse oocytes. Immunoblot analysis showed that RASSF1A is constantly expressed at all stages of oocytes during meiotic maturation (Figure 1A). Subsequently, we investigated subcellular localization of RASSF1A during meiotic maturation. Because of lack of specific antibodies detecting endogenous mouse RASSF1A, we injected a low concentration of mRNAs encoding human RASSF1A-mCherry and visualized exogenous human RASSF1A. At the germinal vesicle (GV) stage, RASSF1A signals were dispersed throughout the cytoplasm as distinct fragments. Upon GV breakdown (GVBD), as the chromatin was condensed into individual chromosomes, RASSF1A began to aggregate around the condensing chromosomes, simultaneous with the newly formed spindle microtubules. At metaphase I (MI) and metaphase II (MII), RASSF1A accumulated at the meiotic spindle microtubules (Figure 1B). To clarify the correlation between RASSF1A and spindle microtubules, we treated MI oocytes with spindle perturbing drugs, taxol and nocodazole. RASSF1A was enriched around the spindle after taxol-induced microtubule polymerization. In contrast, RASSF1A was not observed in the spindle microtubules after disassembling microtubules with nocodazole (Figure 1C). Our results suggest that RASSF1A is involved in spindle formation and/or organization during meiotic maturation.

### Knockdown of RASSF1A Perturbs Polar Body Extrusion by Impairing Spindle Organization and Chromosome Alignment

**FIGURE 2:**
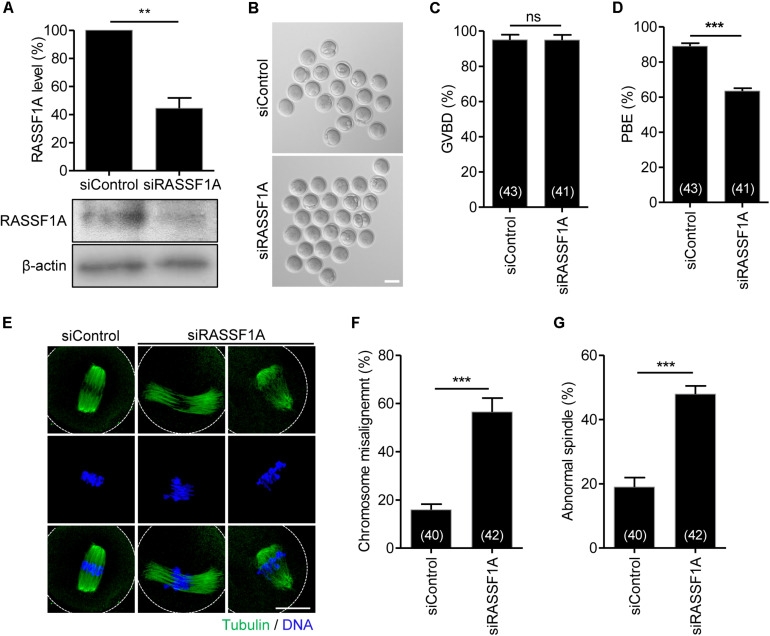
Knockdown of RASSF1A perturbs polar body extrusion by impairing spindle organization and chromosome alignment. **(A)** GV oocytes injected with RASSF1A siRNA were cultured for 24 h in the presence of IBMX. Knockdown of RASSF1A was confirmed by immunoblot analysis. β-actin was used as a loading control. Each lane contains 50 oocytes. Level of RASSF1A expression was quantified and expressed as the mean ± SEM from two independent experiments. **p < 0.001. **(B–D)** RASSF1A knockdown oocytes were cultured in IBMX-free medium for 13 h, and the rates of GVBD and polar body extrusion (PBE) were scored and are shown with representative images. Scale bar, 80 μM. The number of oocytes analyzed is specified in brackets. Data are presented as mean ± SEM from three independent experiments. ns, not significant, ***p < 0.0001. **(E–G)** RASSF1A knockdown oocytes were fixed at the MI stage and stained with anti-acetylated-α-tubulin antibody and DAPI. Chromosome misalignment and spindle abnormality were quantified and are shown with representative images. Dashed line indicates oocyte cortex. Scale bar, 40 μM. The number of oocytes analyzed is specified in brackets. Data are presented as mean ± SEM from three independent experiments. ***p < 0.0001.

To investigate the function of RASSF1A during meiotic maturation, we injected siRNAs targeting RASSF1A (siRASSF1A) or scrambled siRNA control (siControl) into GV-stage oocytes and cultured the oocytes for 24 h. Immunoblot analysis showed that RASSF1 was successfully knocked down after siRNA injection (Figure 2A). We subsequently assessed meiotic progression in RASSF1A knockdown oocytes. Although RASSF1A knockdown did not affect GVBD, the rate of polar body extrusion was significantly decreased, causing the MI arrest (Figures 2B–D). These results suggest that RASSF1A is not dispensable for progression of meiotic maturation in mouse oocytes.

To investigate the molecular basis of MI arrest after RASSF1A knockdown, we examined spindle and chromosome organization of RASSF1A knockdown oocytes arrested at the MI stage. While most control oocytes showed typical barrel-shaped spindles with well-aligned chromosomes at the metaphase plate, a number of RASSF1A knockdown oocytes displayed abnormal spindle organization, namely, unusually elongated and/or disorganized spindle. Moreover, chromosomes were unaligned or dispersed on the abnormal spindle in RASSF1A knockdown oocytes (Figures 2E–G).

### RASSF1A Knockdown Impairs Kinetochore-Microtubule Attachment and Increases Aneuploidy

**FIGURE 3:**
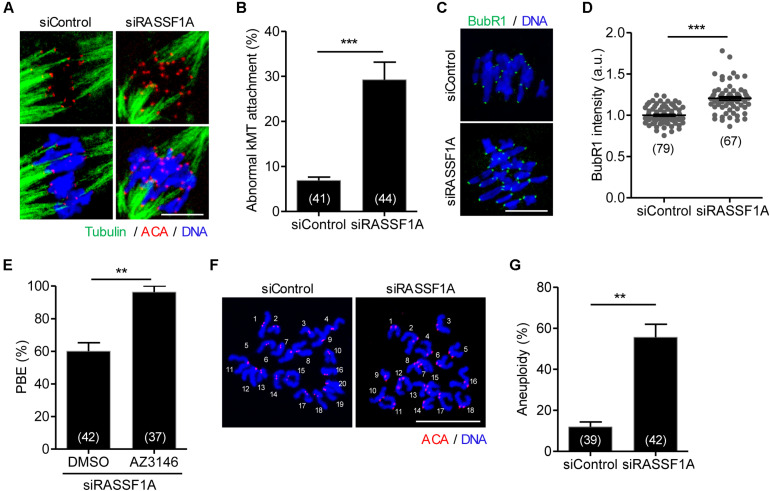
RASSF1A knockdown impairs kinetochore-microtubule attachment and increases aneuploidy. Oocytes injected with siControl or siRASSF1A were cultured in medium containing IBMX for 24 h and then transferred to IBMX-free medium for 10 h. **(A,B)** After cold treatment, oocytes were fixed and stained with anti-centromere antibody (ACA), anti-acetylated-α-tubulin antibody, and DAPI to visualize kinetochore, spindle, and DNA, respectively. Abnormal kinetochore-microtubule (kMT) attachment was quantified and shown with representative images. Scale bar, 10 μM. Data are presented as mean ± SEM from three independent experiments. The number of oocytes analyzed is specified in brackets. ***p < 0.0001. **(C,D)** Oocytes at MI stage (at 10 h of culture after IBMX release) were fixed and stained with anti-BubR1 antibody with DAPI for DNA staining. The intensity of BubR1 was quantified and is shown with representative images. Scale bar, 10 μM. Data are presented as mean ± SEM from three independent experiments. The number of oocytes analyzed is specified in brackets. ***p < 0.0001. **(E)** RASSF1A knockdown oocytes were cultured in the presence of AZ3146 and the PBE rate was scored. The number of oocytes analyzed is specified in brackets and data are presented as mean ± SEM. **p < 0.001. **(F,G)** Chromosome spreading of RASSF1A knockdown MII oocytes. Kinetochore and DNA were stained with ACA and DAPI, respectively. Scale bar, 20 μM. The incidence of aneuploidy was quantified. The number of oocytes analyzed is specified in brackets. Data are presented as mean ± SEM. **p < 0.001.

Given that RASSF1A was colocalized at the spindle microtubules, it is likely that misaligned chromosomes are caused by improper attachment of microtubules to the kinetochore on the chromosomes. To investigate this possibility, we examined kMT attachment after cold treatment to depolymerize unstable microtubules not attached to kinetochores. While kinetochores remained fully attached by spindle microtubules in control oocytes, RASSF1A knockdown oocytes exhibited defective kMT attachments (Figure 3A). The rate of aberrant kMT attachment in RASSF1A knockdown oocytes increased compared to that in the control (Figure 3B).

The findings that RASSF1A knockdown caused MI arrest and impaired proper kMT attachment led us to investigate whether MI arrest by RASSF1A knockdown is caused by activation of SAC. To investigate this, a core protein of SAC complexes, BubR1, was stained and analyzed. Immunostaining analysis revealed that BubR1 level was significantly increased at the kinetochores in RASSF1A knockdown oocytes compared to that in the control (Figures 3C,D). This result suggests that the impaired kMT attachment induced by RASSF1A knockdown activates SAC, which causes MI arrest. Indeed, inhibition of SAC activity with Mps1 kinase inhibitor AZ3146 rescued the MI arrest caused by RASSF1A knockdown (Figure 3E).

Because proper kMT attachments are essential to ensure faithful chromosome segregation, we assumed that RASSF1A knockdown causes chromosome missegregation that eventually leads to aneuploidy. To confirm this possibility, we performed karyotype analysis of MII oocytes by chromosome spreading combined with kinetochore labeling. As expected, the incidence of aneuploidy was significantly increased after RASSF1A knockdown (Figures 3F,G). Therefore, our results suggest that RASSF1A is required for proper kMT attachment, thereby ensuring proper chromosome segregation during oocyte meiosis.

### RASSF1A Regulates α-Tubulin Acetylation in Mouse Oocytes

**FIGURE 4:**
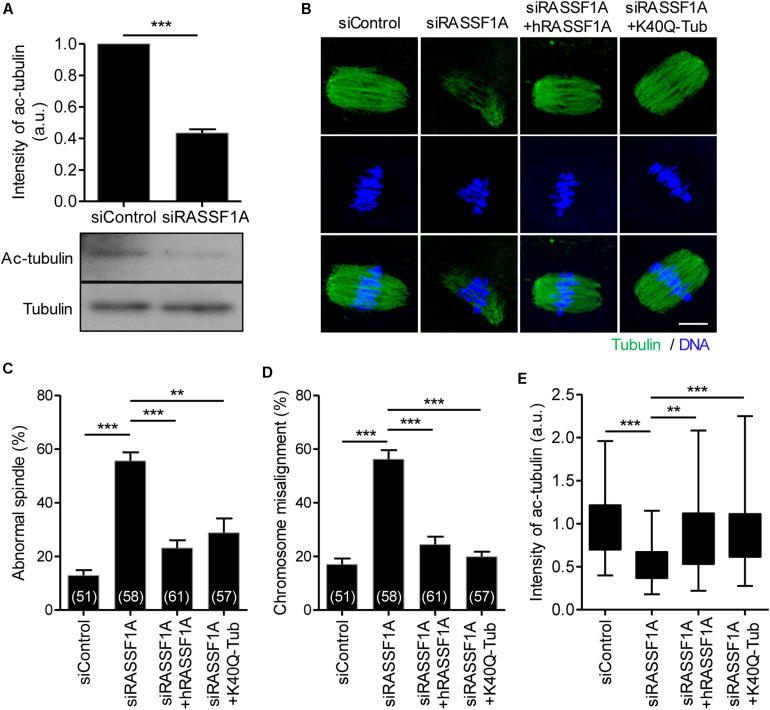
RASSF1A regulates α-tubulin acetylation in mouse oocytes. **(A)** Level of acetylated α-tubulin in control and RASSF1A knockdown oocytes was examined by immunoblot analysis and quantified. Data are presented as mean ± SEM from two independent experiments. ***p < 0.0001. **(B–E)** Oocytes injected with siControl, siRASSF1A, siRASSF1A + hRASSF1A, or siRASSF1A + K40Q α-tubulin were cultured for 10 h in IBMX-free medium and stained with anti-acetylated-α-tubulin antibody and DAPI for spindle and chromosome staining, respectively. Spindle abnormality, chromosome misalignment, and intensity of acetylated α-tubulin were quantified and shown with representative images. Scale bar, 20 μM. Data are presented as mean ± SEM from three independent experiments. The number of oocytes analyzed is specified in brackets. **p < 0.001, ***p < 0.0001.

It is widely reported that microtubule stability is regulated by the acetylation of α-tubulin on K40 residue. Because RASSF1A knockdown impairs spindle organization, we examined the expression level of acetylated α-tubulin in RASSF1A knockdown oocytes. Interestingly, the level of acetylated α-tubulin was significantly decreased after RASSF1A knockdown (Figure 4A). To validate the RASSF1A-mediated change of α-tubulin acetylation, we attempted to rescue the phenotypes in RASSF1A knockdown oocytes by overexpressing human RASSF1A (hRASSF1A) and stained oocytes with anti-α-acetylated tubulin antibody. Notably, spindle and chromosome configuration and level of α-tubulin acetylation were restored after hRASSF1A overexpression (Figures 4B–E). More importantly, the phenotypes were also rescued by overexpressing acetylation-mimicking K40Q α-tubulin rather than hRASSF1A (Figures 4B–E). This result implies that the downstream target of RASSF1A is likely to be a K40 acetylation of α-tubulin.

### SIRT2 and HDAC6 Are the Downstream Targets of RASSF1A in Oocytes

**FIGURE 5:**
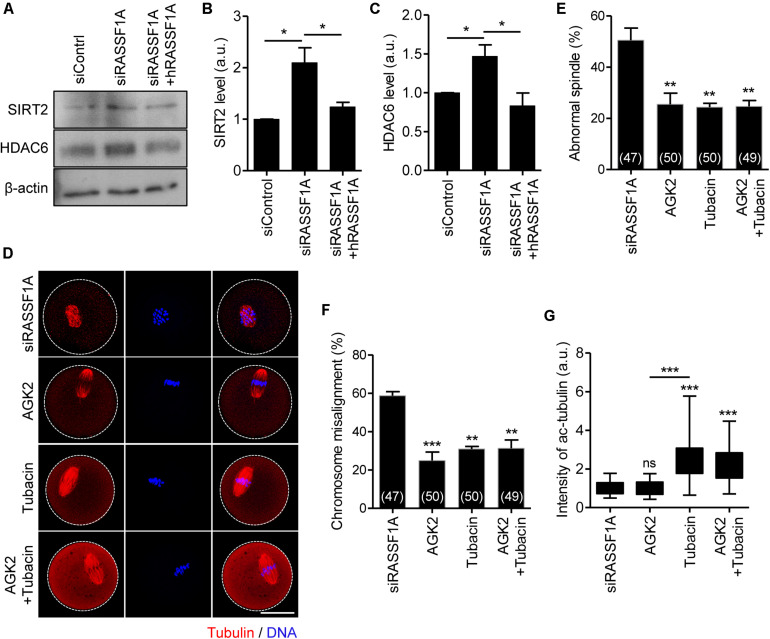
SIRT2 and HDAC6 are the downstream targets of RASSF1A during meiotic maturation in mouse oocytes. **(A)** Immunoblot analysis of SIRT2 and HDAC6 in oocytes injected with siControl, siRASSF1A, or siRASSF1A + hRASSF1A. **(B,C)** Levels of SIRT2 and HDAC6 were quantified. Data are presented as mean ± SEM from two independent experiments. *p < 0.05. **(D–G)** RASSF1A knockdown oocytes were treated with either 5 μM AGK2 or 2 μM tubacin for 3 h after 5 h culture in IBMX-free medium and stained with anti-acetylated-α-tubulin antibody and DAPI for spindle and chromosome staining, respectively. Spindle abnormality, chromosome misalignment, and intensity of acetylated α-tubulin were quantified and are shown with representative images. Dashed line indicates oocyte cortex. Scale bar, 40 μM. Data are presented as mean ± SEM from three independent experiments. The number of oocytes analyzed is specified in brackets. ns, not significant, **p < 0.001, ***p < 0.0001.

To dissect the link between RASSF1A and α-tubulin acetylation, we examined the protein expression of SIRT2 and HDAC6, which are related to deacetylation of α-tubulin (Hubbert et al., 2002; North et al., 2003; Zhang et al., 2014). Immunoblot analysis showed that both SIRT2 and HDAC6 levels were increased after RASSF1A knockdown but rescued by hRASSF1 overexpression (Figures 5A–C). This result suggests that RASSF1A is required to suppress SIRT2 and HDAC6 levels in mouse oocytes. To further clarify the association of SIRT2 and HDAC6 in regulation of RASSF1A-mediated α-tubulin acetylation, we treated RASSF1A knockdown oocytes with either AGK2 (SIRT2 inhibitor) or tubacin (HDAC6 inhibitor). To rule out the possible effects of inhibitors on GVBD, we treated oocytes with inhibitors after 5 h release from IBMX (corresponding to pro-MI stage) and MI oocytes were fixed after 3 h later and subsequently stained with anti-α-acetylated tubulin antibody. Interestingly, defects in spindle and chromosome organization were rescued by AGK2 or tubacin treatment (Figures 5D–F). Moreover, the levels of α-tubulin acetylation were increased by tubacin, but not by AGK2 (Figures 5D,G). However, combined treatment of AGK2 and tubacin was not able to increase the efficiency of rescue in spindle and chromosome defects as well as α-tubulin acetylation (Figures 5D–G). Taken together, our results suggest that SIRT2 and HDAC6 are *bona fide* downstream targets of RASSF1A in oocytes.

## Discussion

In this study, we explored the expression, subcellular localization, and potential role of RASSF1A during meiotic maturation in mouse oocytes. We found that RASSF1A is localized at spindle microtubules and regulates spindle organization and chromosome segregation by modulating α-tubulin acetylation via SIRT2 and HDAC6 in mouse oocytes.

In somatic cells, RASSF1A associates with microtubules and localizes to the centrosome and spindle during mitosis (Liu et al., 2003; Dallol et al., 2004; Vos et al., 2004; Donninger et al., 2007). Consistent with this, our data show that RASSF1A is colocalized with spindle microtubules during oocyte meiosis, suggesting a conserved role of RASSF1A in regulation of spindle microtubules during both mitosis and meiosis. In support of this observation, RASSF1A knockdown in oocytes disrupts spindle assembly and chromosome alignment. Given that correct chromosome alignment to the metaphase plate requires bipolar attachment and tension between kinetochore and microtubules, it is conceivable that spindle and chromosome abnormalities in RASSF1A knockdown oocytes are a consequence of impaired kMT attachment, which is the leading cause of chromosome missegregation. Consistent with this notion, the rate of aberrant kMT attachment and the incidence of aneuploidy significantly increased after RASSF1A knockdown in oocytes. In the presence of unattached kinetochores, SAC proteins form a complex that inactivates anaphase promoting complex/cyclosome (APC/C) by inhibiting Cdc20 (Tauchman et al., 2015). Given that RASSF1A knockdown significantly increased impaired kMT attachment, it is likely that the increase in proportion of MI-arrested oocytes after RASSF1A knockdown is due to SAC activation. Indeed, we found that the BubR1 signal persists at the kinetochore of metaphase chromosomes in RASSF1A knockdown oocytes compared with controls. Moreover, MI arrest by RASSF1A knockdown was rescued by blocking kinetochore recruitment of SAC components using Mps1 kinase inhibitor (Abrieu et al., 2001). Therefore, our data suggest that RASSF1A knockdown activates SAC and thereby prevents anaphase onset during oocyte meiosis. In addition, RASSF1A has been found to act as an inhibitor of APC/C-Cdc20 during mitosis and this inhibition is relieved by RASSF1A phosphorylation (Song et al., 2004, 2009; Chow et al., 2012). Thus, RASSF1A itself has activity to prevent anaphase onset, but depletion of RASSF1A disrupts spindle organization which in turn activates SAC and inhibits anaphase onset.

In addition to spindle localization, RASSF1A was detected as distinct fragments in the cytoplasm of oocytes. Given that RASSF1A contains a Ras association domain and is potentially an effector of the Ras oncogene and that Ras is associated with the endomembrane system (Choy et al., 1999; Vos et al., 2000; Rodriguez-Viciana et al., 2004), the cytoplasmic signal of RASSF1A seems to be associated with Ras signaling in mouse oocytes. Consistent with this, Golgi is observed as distinct fragments dispersed throughout the cytoplasm in mouse oocytes (Payne and Schatten, 2003). In this regard, it is tempting to speculate that the RASSF1A knockdown phenotype observed in this study is associated with impaired Ras signaling in oocytes. However, this is less likely because interference of Ras signaling in mouse oocytes by microinjecting antisense oligonucleotide or anti-Ras monoclonal antibody had no effect on meiotic maturation (Yamauchi et al., 1994).

Previous studies have shown that acetylated α-tubulin is essential for microtubule dynamics in mouse oocytes (de Pennart et al., 1988; Schatten et al., 1988). Like other post-translational modifications of tubulin, acetylation of α-tubulin is under the control of balanced enzyme activities. In mouse oocytes, it is primarily catalyzed by the acetyltransferase αTAT and reversed by the deacetylases HDAC6 and SIRT2 (Hubbert et al., 2002; North et al., 2003; Zhang et al., 2014; Coombes et al., 2016). In line with this, we found a decrease in α-tubulin acetylation with concomitant increase in SIRT2 and HDAC6 levels in oocytes after RASSF1A knockdown. Importantly, the spindle and chromosome defects in RASSF1A knockdown oocytes were restored by overexpressing K40Q tubulin. Furthermore, either SIRT2 or HDAC6 inhibition could also rescue RASSF1A knockdown phenotypes. These data lead us to propose that RASSF1A knockdown increases SIRT2 and HDAC6 levels, which in turn promote tubulin deacetylation, compromising microtubule stability in oocytes. The reduced microtubule stability impairs kMT attachment, leading to spindle and chromosome abnormalities and aneuploidy. Given that microtubule dynamics can be modulated by a series of microtubule-associated proteins (MAPs) that bind directly to tubulin, and that RASSF1A could associate with microtubules via MAPs (Song et al., 2005; Halpain and Dehmelt, 2006), it is possible to speculate that RASSF1A acts as a masking protein that suppresses SIRT2 and HDAC6 recruitment and stabilization on spindle microtubules during meiotic maturation in oocytes. In this respect, we assumed that RASSF1A knockdown allows SIRT2 and HDAC6 to bind to microtubules, which increases stability of SIRT2 and HDAC6. Given that the acetylation level of α-tubulin also increases after HDAC3 depletion in mouse oocytes (Li et al., 2017), our data could not exclude the possibility that RASSF1A regulates α-tubulin acetylation via targets other than SIRT2 and HDAC6 in oocytes. We also could not rule out the possibility of SIRT2 and HDAC6 modulations by regulators other than RASSF1A in mouse oocytes. Further studies are required to clarify these issues.

## Conclusion

In conclusion, our results show that RASSF1A is essential for spindle organization and chromosome alignment by modulating tubulin acetylation via SIRT2 and HDAC6 during meiotic maturation in oocytes.

## Data Availability Statement

The original contributions presented in the study are included in the article/supplementary materials, further inquiries can be directed to the corresponding author.

## Ethics Statement

The animal study was reviewed and approved by the Institutional Animal Care and Use Committees of Sungkyunkwan University (approval ID: SKKUIACUC2 019-04-28-4).

## Author Contributions

H-JJ conceived the project and performed all experiments. H-JJ and JO designed and analyzed experiments and wrote the manuscript. JO supervised the study. All authors contributed to the article and approved the submitted version.

## Conflict of Interest

The authors declare that the research was conducted in the absence of any commercial or financial relationships that could be construed as a potential conflict of interest.

## References

[B1] Abrieu A. Magnaghi-Jaulin L. Kahana J. A. Peter M. Castro A. Vigneron S. (2001). Mps1 is a kinetochore-associated kinase essential for the vertebrate mitotic checkpoint. *Cell* 106 83–93. 10.1016/S0092-8674(01)00410-X 11461704

[B2] Bennabi I. Terret M. E. Verlhac M. H. (2016). Meiotic spindle assembly and chromosome segregation in oocytes. *J. Cell Biol.* 215 611–619. 10.1083/jcb.201607062 27879467 PMC5147004

[B3] Burbee D. G. Forgacs E. Zochbauer-Muller S. Shivakumar L. Fong K. Gao B. (2001). Epigenetic inactivation of RASSF1A in lung and breast cancers and malignant phenotype suppression. *J. Natl. Cancer Inst.* 93 691–699. 10.1093/jnci/93.9.691 11333291 PMC4374741

[B4] Chan M. W. Chan L. W. Tang N. L. Lo K. W. Tong J. H. Chan A. W. (2003). Frequent hypermethylation of promoter region of RASSF1A in tumor tissues and voided urine of urinary bladder cancer patients. *Int. J. Cancer* 104 611–616. 10.1002/ijc.10971 12594816

[B5] Chow C. Wong N. Pagano M. Lun S. W. Nakayama K. I. Nakayama K. (2012). Regulation of APC/CCdc20 activity by RASSF1A-APC/CCdc20 circuitry. *Oncogene* 31 1975–1987. 10.1038/onc.2011.372 21874044 PMC3325600

[B6] Choy E. Chiu V. K. Silletti J. Feoktistov M. Morimoto T. Michaelson D. (1999). Endomembrane trafficking of ras: the CAAX motif targets proteins to the ER and Golgi. *Cell* 98 69–80. 10.1016/S0092-8674(00)80607-8 10412982

[B7] Coombes C. Yamamoto A. Mcclellan M. Reid T. A. Plooster M. Luxton G. W. (2016). Mechanism of microtubule lumen entry for the alpha-tubulin acetyltransferase enzyme alphaTAT1. *Proc. Natl. Acad. Sci. U S A* 113 E7176–E7184. 10.1073/pnas.1605397113 27803321 PMC5135325

[B8] Dallol A. Agathanggelou A. Fenton S. L. Ahmed-Choudhury J. Hesson L. Vos M. D. (2004). RASSF1A interacts with microtubule-associated proteins and modulates microtubule dynamics. *Cancer Res.* 64 4112–4116. 10.1158/0008-5472.CAN-04-0267 15205320

[B9] Dammann R. Li C. Yoon J. H. Chin P. L. Bates S. Pfeifer G. P. (2000). Epigenetic inactivation of a RAS association domain family protein from the lung tumour suppressor locus 3p21.3. *Nat. Genet.* 25 315–319. 10.1038/77083 10888881

[B10] de Pennart H. Houliston E. Maro B. (1988). Post-translational modifications of tubulin and the dynamics of microtubules in mouse oocytes and zygotes. *Biol. Cell* 64 375–378. 2906552 10.1016/0248-4900(88)90012-3

[B11] Desai A. Mitchison T. J. (1997). Microtubule polymerization dynamics. *Annu. Rev. Cell Dev. Biol.* 13 83–117. 10.1146/annurev.cellbio.13.1.83 9442869

[B12] Donninger H. Vos M. D. Clark G. J. (2007). The RASSF1A tumor suppressor. *J. Cell Sci.* 120 3163–3172. 10.1242/jcs.010389 17878233

[B13] Halpain S. Dehmelt L. (2006). The MAP1 family of microtubule-associated proteins. *Gen. Biol.* 7:224 10.1186/gb-2006-7-6-224 PMC1779536 16938900

[B14] Hubbert C. Guardiola A. Shao R. Kawaguchi Y. Ito A. Nixon A. (2002). HDAC6 is a microtubule-associated deacetylase. *Nature* 417 455–458. 10.1038/417455a 12024216

[B15] Jung H. Y. Jung J. S. Whang Y. M. Kim Y. H. (2013). RASSF1A Suppresses Cell Migration through Inactivation of HDAC6 and Increase of Acetylated alpha-Tubulin. *Cancer Res. Treat.* 45 134–144. 10.4143/crt.2013.45.2.134 23864847 PMC3710963

[B16] Li L. Yang X. J. (2015). Tubulin acetylation: responsible enzymes, biological functions and human diseases. *Cell Mol. Life Sci.* 72 4237–4255. 10.1007/s00018-015-2000-5 26227334 PMC11113413

[B17] Li X. Liu X. Gao M. Han L. Qiu D. Wang H. (2017). HDAC3 promotes meiotic apparatus assembly in mouse oocytes by modulating tubulin acetylation. *Development* 144 3789–3797. 10.1242/dev.153353 28935703

[B18] Liu L. Tommasi S. Lee D. H. Dammann R. Pfeifer G. P. (2003). Control of microtubule stability by the RASSF1A tumor suppressor. *Oncogene* 22 8125–8136. 10.1038/sj.onc.1206984 14603253

[B19] Magiera M. M. Janke C. (2014). Post-translational modifications of tubulin. *Curr. Biol.* 24 R351–R354. 10.1016/j.cub.2014.03.032 24801181

[B20] North B. J. Marshall B. L. Borra M. T. Denu J. M. Verdin E. (2003). The human Sir2 ortholog, SIRT2, is an NAD+-dependent tubulin deacetylase. *Mol. Cell* 11 437–444. 10.1016/S1097-2765(03)00038-8 12620231

[B21] Pan Z. G. Kashuba V. I. Liu X. Q. Shao J. Y. Zhang R. H. Jiang J. H. (2005). High frequency somatic mutations in RASSF1A in nasopharyngeal carcinoma. *Cancer Biol. Ther.* 4 1116–1122. 10.4161/cbt.4.10.2023 16096369

[B22] Payne C. Schatten G. (2003). Golgi dynamics during meiosis are distinct from mitosis and are coupled to endoplasmic reticulum dynamics until fertilization. *Dev. Biol.* 264 50–63. 10.1016/j.ydbio.2003.08.004 14623231

[B23] Pfeifer G. P. Dammann R. (2005). Methylation of the tumor suppressor gene RASSF1A in human tumors. *Biochemistry* 70 576–583. 10.1007/s10541-005-0151-y 15948711

[B24] Rodriguez-Viciana P. Sabatier C. Mccormick F. (2004). Signaling specificity by Ras family GTPases is determined by the full spectrum of effectors they regulate. *Mol. Cell Biol.* 24 4943–4954. 10.1128/MCB.24.11.4943-4954.2004 15143186 PMC416418

[B25] Sadoul K. Khochbin S. (2016). The growing landscape of tubulin acetylation: lysine 40 and many more. *Biochem. J.* 473 1859–1868. 10.1042/BCJ20160172 27354562

[B26] Schatten G. Simerly C. Asai D. J. Szoke E. Cooke P. Schatten H. (1988). Acetylated alpha-tubulin in microtubules during mouse fertilization and early development. *Dev. Biol.* 130 74–86. 10.1016/0012-1606(88)90415-0 3053299

[B27] Shah J. V. Cleveland D. W. (2000). Waiting for anaphase: Mad2 and the spindle assembly checkpoint. *Cell* 103 997–1000. 10.1016/S0092-8674(00)00202-6 11163175

[B28] Song M. S. Chang J. S. Song S. J. Yang T. H. Lee H. Lim D. S. (2005). The centrosomal protein RAS association domain family protein 1A (RASSF1A)-binding protein 1 regulates mitotic progression by recruiting RASSF1A to spindle poles. *J. Biol. Chem.* 280 3920–3927. 10.1074/jbc.M409115200 15546880

[B29] Song M. S. Song S. J. Ayad N. G. Chang J. S. Lee J. H. Hong H. K. (2004). The tumour suppressor RASSF1A regulates mitosis by inhibiting the APC-Cdc20 complex. *Nat. Cell Biol.* 6 129–137. 10.1038/ncb1091 14743218

[B30] Song S. J. Song M. S. Kim S. J. Kim S. Y. Kwon S. H. Kim J. G. (2009). Aurora A regulates prometaphase progression by inhibiting the ability of RASSF1A to suppress APC-Cdc20 activity. *Cancer Res.* 69 2314–2323. 10.1158/0008-5472.CAN-08-3984 19276349

[B31] Strzyz P. (2016). Post-translational modifications: Extension of the tubulin code. *Nat. Rev. Mol. Cell Biol.* 17:609 10.1038/nrm.2016.117 27552972

[B32] Tauchman E. C. Boehm F. J. Deluca J. G. (2015). Stable kinetochore-microtubule attachment is sufficient to silence the spindle assembly checkpoint in human cells. *Nat. Commun.* 6:10036. 10.1038/ncomms10036 26620470 PMC4686653

[B33] Taylor S. S. Scott M. I. Holland A. J. (2004). The spindle checkpoint: a quality control mechanism which ensures accurate chromosome segregation. *Chromosome Res.* 12 599–616. 10.1023/B:CHRO.0000036610.78380.51 15289666

[B34] Thompson S. L. Compton D. A. (2011). Chromosome missegregation in human cells arises through specific types of kinetochore-microtubule attachment errors. *Proc. Natl. Acad. Sci. U S A* 108 17974–17978. 10.1073/pnas.1109720108 21997207 PMC3207692

[B35] Vos M. D. Ellis C. A. Bell A. Birrer M. J. Clark G. J. (2000). Ras uses the novel tumor suppressor RASSF1 as an effector to mediate apoptosis. *J. Biol. Chem.* 275 35669–35672. 10.1074/jbc.C000463200 10998413

[B36] Vos M. D. Martinez A. Elam C. Dallol A. Taylor B. J. Latif F. (2004). A role for the RASSF1A tumor suppressor in the regulation of tubulin polymerization and genomic stability. *Cancer Res.* 64 4244–4250. 10.1158/0008-5472.CAN-04-0339 15205337

[B37] Yamauchi N. Kiessling A. A. Cooper G. M. (1994). The Ras/Raf signaling pathway is required for progression of mouse embryos through the two-cell stage. *Mol. Cell Biol.* 14 6655–6662. 10.1128/MCB.14.10.6655 7935384 PMC359195

[B38] Zhang L. Hou X. Ma R. Moley K. Schedl T. Wang Q. (2014). Sirt2 functions in spindle organization and chromosome alignment in mouse oocyte meiosis. *FASEB J.* 28 1435–1445. 10.1096/fj.13-244111 24334550 PMC3929683

